# INTEGRATING ACUTE AND LONG-TERM SERVICES: DOES IT WORK, CAN IT WORK, WILL IT WORK?

**DOI:** 10.1093/geroni/igz038.848

**Published:** 2019-11-08

**Authors:** Robert A Applebaum, Jennifer Heston

**Affiliations:** 1 Miami University, Scripps Gerontology Center, Oxford, Ohio, United States; 2 Miami University Scripps Geron, OXFORD, Ohio, United States

## Abstract

The expansion of managed long-term services and supports has generated considerable interest over the last decade. However, studies on the impact of these efforts have produced mixed findings. Additionally, there is limited information about the care management models used in implementation. This lack of data makes it impossible to assess whether differences in managed care plan approaches have an impact on participants. Our study sought to gain better understanding of the integrated care management models being implemented in Ohio’s MyCare Demonstration. Through qualitative interviews with 50 respondents, including area agency care managers, managed care staff, and service providers, we documented strengths and weaknesses of one integrated care management model used in Ohio’s demonstration. Understanding what is inside the black box of managed care/care management model implementation is key to gaining insights into whether such an approach can ultimately improve the health and long-term service systems for older people with disability.

